# MDFI promotes the proliferation and tolerance to chemotherapy of colorectal cancer cells by binding ITGB4/LAMB3 to activate the AKT signaling pathway

**DOI:** 10.1080/15384047.2024.2314324

**Published:** 2024-02-20

**Authors:** Ding Ma, Shuwen Liu, Kua Liu, Lingkai Kong, Lingjun Xiao, Qilei Xin, Chunping Jiang, Junhua Wu

**Affiliations:** a State Key Laboratory of Pharmaceutical Biotechnology, National Institute of Healthcare Data Science at Nanjing University, Jiangsu Key Laboratory of Molecular Medicine, Medical School, Nanjing University, Nanjing, Jiangsu, China; b Department of Gastroenterology, Third Xiangya Hospital, Central South University, Changsha, Hunan, China; c Jinan Microecological Biomedicine Shandong Laboratory, Shounuo City Light West Block, Jinan City, Shandong Province, China

**Keywords:** scRNA-seq (single-cell RNA sequencing), PPIs (protein-protein interactions), MDFI (MyoD family inhibitor), ITGB4/LAMB3, AKT

## Abstract

Colorectal cancer (CRC) is one of the most lethal cancers. Single-cell RNA sequencing (scRNA-seq) and protein-protein interactions (PPIs) have enabled the systematic study of CRC. In our research, the activation of the AKT pathway in CRC was analyzed by KEGG using single-cell sequencing data from the GSE144735 dataset. The correlation and PPIs of MDFI and ITGB4/LAMB3 were examined. The results were verified in the TCGA and CCLE and further tested by coimmunoprecipitation experiments. The effect of MDFI on the AKT pathway via ITGB4/LAMB3 was validated by knockdown and lentiviral overexpression experiments. The effect of MDFI on oxaliplatin/fluorouracil sensitivity was probed by colony formation assay and CCK8 assay. We discovered that MDFI was positively associated with ITGB4/LAMB3. In addition, MDFI was negatively associated with oxaliplatin/fluorouracil sensitivity. MDFI upregulated the AKT pathway by directly interacting with LAMB3 and ITGB4 in CRC cells, and enhanced the proliferation of CRC cells via the AKT pathway. Finally, MDFI reduced the sensitivity of CRC cells to oxaliplatin and fluorouracil. In conclusion, MDFI promotes the proliferation and tolerance to chemotherapy of colorectal cancer cells, partially through the activation of the AKT signaling pathway by the binding to ITGB4/LAMB3. Our findings provide a possible molecular target for CRC therapy.

## Introduction

Colorectal cancer (CRC) is one of the most common malignant digestive tract cancers in the world. CRC accounts for approximately 1/10 of all tumor deaths.^1^ An in-depth analysis of the potential mechanism of CRC occurrence and development is urgent and essential for the development of effective therapeutic targets and new clinical treatment strategies.

Single-cell RNA sequencing (scRNA-seq) has enabled the systematic study of all kinds of human diseases, especially for tumors.^2,3^ Wang et al. discovered that cancer epithelial cells are important in tumor CRC progression, and key targets correlated with the prognosis of CRC have been found via single-cell RNA sequencing (scRNA-seq).^4^

The molecular landscapes and phenotypes of CRC have been revealed through extensive multiomics studies.^5,6^ The scRNA-seq method revealed the dynamic relationships among cellular components and the diversity of their functions, and CRC molecular subtypes were determined by scRNA-seq.^7,8^ To reveal an unbiased mechanism resulting in CRC tumorigenesis and development, we performed detailed analyses of scRNA-seq data from the GSE144735 dataset. The differentially expressed genes between normal epithelial cells and tumors were identified. Pathway analyses were then performed to identify pathways that were enriched in DEGs, after which we used WGCNA to identify the gene group (module) that had the strongest pathway correlation. Finally, we identified the unbiased relationship between the pathway and the genes. The results revealed that the PI3K-AKT pathway was active in tumor cells and that MDFI upregulated the PI3K-AKT pathway by directly regulating LAMB3 and ITGB4.

MDFI is an inhibitor of the MyoD family. It has a highly conserved carboxyl terminal functional domain, the I-mfa domain (an inhibitor of the MyoD family a). As an important myogenic inhibitory protein in early embryonic development, MDFI is generally considered to regulate the development of biological segments by inhibiting myogenesis through two mechanisms: 1. Shielding the nuclear localization signal of the MyoD family and maintaining it in the cytoplasm; 2. Regulating the function of myogenic transcription factors in cells and inhibiting DNA binding activity.^9^ With the continuous progression of MDFI research, MDFI was found to have significantly higher expression in pancreatic cancer, hepatocellular carcinoma, prostate cancer and ovarian cancer tissues than in adjacent normal tissues.^10,11^ In CRC, the MDFI promoter is highly methylated, which is a key event in tumorigenesis and progression.^12^ Wu T et al. confirmed that MDFI is closely related to drug action in breast cancer.^13^ However, the role of the MDFI in CRC has not been fully elucidated.

Coant N et al. demonstrated that AKT signaling accelerates the progression of the cell cycle and promotes cell proliferation in CRC.^14^ It was further confirmed that activation of the AKT signaling pathway in CRC led to increased CCND1 protein expression.^15^ Mutations in upstream regulators, such as ITGB4 and LAMB3, are often responsible for abnormal activation of AKT signaling pathways. Abnormal expression of ITGB4 and LAMB3 has been proven to be a marker of poor prognosis in CRC patients, and ITGB4 and LAMB3 overexpression induces the activation of the AKT pathway and promotes cell proliferation, tumor growth and metastasis in CRC.^16–18^ Leng et al. discovered that ITGB4 activated the AKT pathway by binding FAK.^18^ LAMB3 is an extracellular matrix protein (ECM),^19^ which is also known to interact with integrins and further regulate the AKT pathway.^20^ Zhang et al. also found that inhibition of LAMB3 suppresses PI3K/Akt signaling pathway activation.^21^ However, the regulatory mechanism of ITGB4 and LAMB3 in CRC is still vague.

In this study, bioinformatics analysis and biological experiments confirmed that MDFI promoted CRC progression and hindered the antitumor activity of chemotherapeutic drugs in CRC. The knockdown of MDFI in CRC significantly inhibited proliferation, induced cell cycle arrest and restored the therapeutic efficacy of 5-fluorouracil (5-FU) and oxaliplatin in CRC. We further confirmed that direct interactions between MDFI and LAMB3 and between MDFI and ITGB4 mediate the AKT pathway. These results help us better understand the cancer-promoting characteristics of the MDFI gene, overcome the limitations of CRC treatment and develop effective treatment regimens.

## Results

### Differences between tumor epithelial cells and normal epithelial cells in CRC

**Figure 1.**
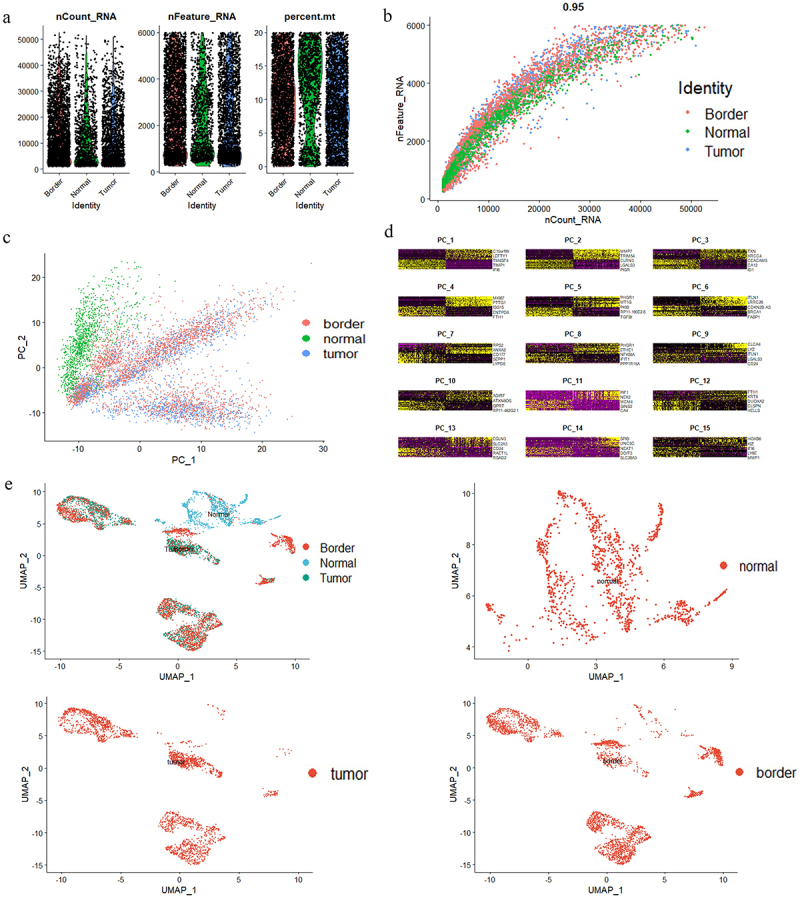
Identification information of epithelial cells based on scRNA‐seq data. A. nCount, nFeature and percent.Mt in colorectal epithelial cells (*n* = 6168). B. The relationship between nCount and nFeature in colorectal epithelial cells. C-D. PCA discriminated normal (*n* = 1144), tumor (*n* = 2212) and border (*n* = 2812) colorectal epithelial cells. E. UMAP representation of the colorectal epithelial cell landscape. Upper left: total epithelial cells. Upper right: matched normal mucosal epithelial cells. Lower left: tumor epithelial cells. Lower right: border epithelial cells.

The most prevalent forms of cancer are of epithelial origin. We obtained scRNA-seq data from gse144735, which contains 27,414 cells from 6 Belgian patients with CRC.^8^ We also obtained an annotation dataset from gse144735 (Table S1). Then, we selected epithelial cells based on the annotation dataset (Table S1), and 6168 epithelial cells were analyzed. All percent.mt (the percentage of mitochondrial genes) were below 20% in cells from the tumor group, the normal (matched normal mucosa) group, and the border group (Figure 1a), which indicated that the cells were not strongly disrupted. The nFeature (the number of genes present in the sample) and nCount (the total UMI count) data were plotted for each sequenced cell (Figure 1a). The three groups were similar with respect to nCount and nFeature, which demonstrated the reliability of the data (Figure 1b).

To classify the cells, we performed PCA dimensionality reduction (Figure 1c–d) and then applied the uniform manifold approximation and projection (UMAP) dimensional reduction technique to cluster the cells (Figure 1e). We discovered that cells from the tumor and matched normal mucosa could be separated by UMAP (Figure 1e). The border had both tumor and matched normal mucosa. Therefore, in the follow-up study, to identify DEGs in CRC, we examined the gene pool between tumor epithelial cells and normal epithelial cells.

### Activation of the PI3K-AKT pathway in CRC epithelial cells

We divided the normal and tumor epithelial cells based on the annotation dataset and obtained the expression matrices of 3356 cells. Differential gene expression analysis was performed using Seurat,^22^ and 715 genes were found to be differentially expressed (Table S2). We performed KEGG analysis to further understand the functions, pathways and upstream regulators of the differentially expressed genes. We discovered that the PI3K-AKT signaling pathway, which is closely associated with the development of tumors, was activated in tumor cells (Figure S1A). All PI3K-AKT signaling pathway-associated genes were identified via the KEGG dataset. In CRC tumor tissues, 14 genes associated with the PI3K-AKT signaling pathway were significantly overexpressed compared with those in normal tissues (Figure S1B-S1C, Table 1).

**Table 1.** Changes in the expression of PI3K-AKT pathway genes in tumor and normal colorectal epithelial cells.

gene	p_val	avg_logFC	pct.tumor	pct.normal
COL1A1	1.43E–133	0.77424	.459	.044
ITGB4	8.40E–51	0.66919	.502	.309
COL1A2	2.74E–103	0.626934	.43	.073
HSP90AA1	1.15E–59	0.60432	.852	.77
COL6A1	1.05E–91	0.554993	.339	.027
DDIT4	2.50E–28	0.488327	.483	.335
SPP1	4.28E–76	0.486835	.259	.003
RHEB	1.16E–38	0.413941	.557	.383
ATF4	3.34E–19	0.390848	.594	.53
LAMB3	6.68E–45	0.358764	.43	.201
ITGA3	1.12E–29	0.357098	.386	.225
EPHA2	2.54E–36	0.329499	.298	.113
ITGB1	5.92E–25	0.270036	.398	.248
HSP90AB1	8.88E–06	0.325112	.786	.71

### The expression of the 420 genes was correlated with the expression pattern of the AKT pathway associated genes in CRC

We chose the top 5000 variant genes for WGCNA in normal and tumor epithelial cells, and we set the power of β = 7as the soft‐thresholding (scale‐free R^2^ = 0.85) (Figure S2A-S2B). We further divided the genes into four modules based on average linkage hierarchical clustering (Figure S2C). Among these modules, the gray module was strongly correlated with most of the PI3K-AKT signaling pathway-associated genes (COL1A1, ITGB4, COL1A2, COL6A1, DDIT4, SPP1, LAMB3, ITGA3, EPHA2 and ITGB1) and tumor stage (Figure S2D). A total of 420 genes in the gray module were used for further analysis (Table S3).

### MDFI regulated the AKT pathway by directly interacting with LAMB3 and ITGB4 in CRC

**Figure 2.**
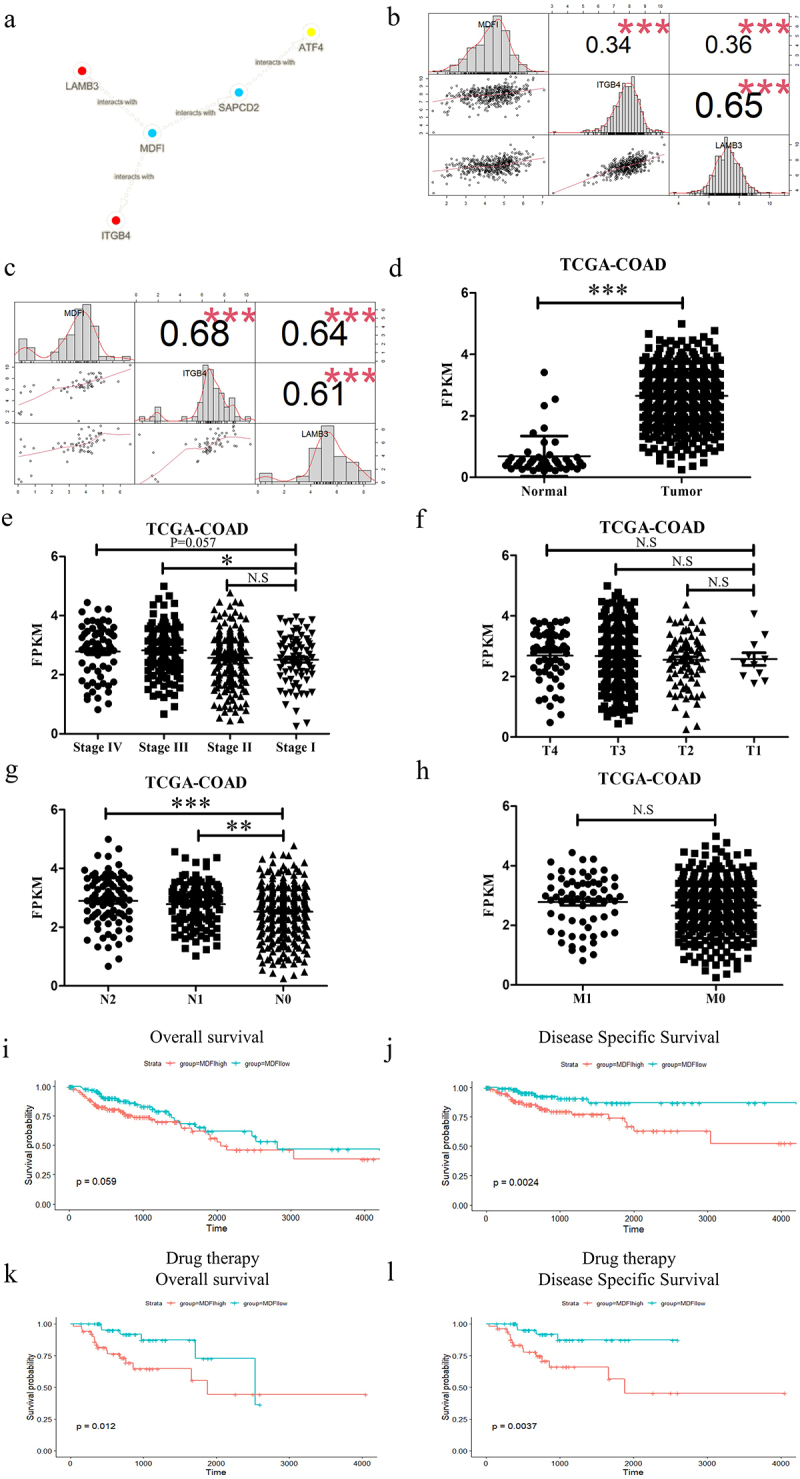
The PI3K-AKT signaling pathway and malignant phenotype regulation by MDFI in colorectal epithelial cells. A. The contact details of MDFI. B. The correlation between the expression of MDFI and the expression of ITGB4 and LAMB3 in TCGA-COAD (*n* = 462). C. The correlation between the expression of MDFI and the expression of ITGB4 and LAMB3 in CCLE (*n* = 57). D. The expression of MDFI (mean with SD) between tumor tissue (*n* = 470) and normal tissue (*n* = 44) in TCGA-COAD cohort. E. The expression of MDFI (mean with SD) in patients with different stages (stage I = 78, stage II = 182, stage III = 131, stage Ⅳ = 65). F. The expression of MDFI (mean with SD) in patients with different T stages (T1 = 11, T2 = 80, T3 = 318, T4 = 58). G. The expression of MDFI (mean with SD) in patients with different N stages (N0 = 277, N1 = 107, N2 = 84). H. The expression of MDFI (mean with SD) in patients with different M stages (M0 = 344, M1 = 65). I. Overall survival (OS) of MDFI in TCGA-COAD (*n* = 458). J. Disease free survival (DFS) of MDFI in TCGA-COAD (*n* = 458). K. Overall survival (OS) of MDFI in 154 patients treated with chemotherapy in TCGA-COAD. L. Disease-free survival (DFS) of MDFI in 154 patients treated with chemotherapy in TCGA-COAD.

To find the direct relationship between 420 genes in the gray module and PI3K-AKT signaling pathway associated genes, we obtained help from human binary protein interactions (HuRIs). We calculated the level 1, level 2, and level 3 counts (see Methods for details) between genes in the gray module and PI3K-AKT pathway associated genes (Table S4). The sorting was done by the sum of level 1, level 2 and level 3 counts, with the MDFI ranked as number 1 (Table S4). MDFI can directly contact LAMB3 and ITGB4 (Figure 2a). Therefore, MDFI is a potential gene that can influence the PI3K-AKT pathway.

Our findings were confirmed by using the validation dataset TCGA and the CCLE dataset. MDFI expression was clearly correlated with LAMB3 and ITGB4 expression (Figure 2b). We also found that the expression of MDFI in the colorectal cell line was closely associated with that of ITGB4 and LAMB3 (Figure 2c).

### The MDFI is correlated with tumour malignancy, as indicated by the Ann Arbor stage, survival time and chemotherapy sensitivity in CRC

The TCGA database showed that MDFI gene expression levels were upregulated in colorectal tumor tissues compared with those in normal tissues (Figure 2d). Subsequently, we analyzed the clinical manifestations of CRC patients and found that CRC patients’ MDFI expression levels were positively correlated with their Ann Arbor stage (Figure 2e–h). The MDFI reduced disease-specific survival (Figure 2i–j). Lymph node metastasis was detected by HE staining. Then, the chi-square test was used to analyze the data of HE-stained CRC samples from the TCGA (*n* = 260). There was a significant positive correlation between lymph node HE staining positivity and MDFI expression. Tissues with high MDFI expression had a higher proportion of positive HE staining (Table 2).

**Table 2.** Pearson chi-square test between positive lymph nodes and the expression of MDFI.

Group	Lymph node metastasis detected by HE staining		Pearson chi-square	p value
	Positive	Negative		
MDFI high	66	64	6.933	0.008
MDFI low	45	85		

The TCGA database showed that chemotherapy is an important treatment for colorectal cancers (Table S5). Of the 154 patients treated with chemotherapy, MDFI reduced overall survival and disease-specific survival (Figure 2k–l). These results suggested that CRC patients treated with chemotherapy and with high MDFI expression have shorter survival and poorer prognosis than CRC patients with low MDFI expression. Therefore, CRC prognosis is significantly affected by the MDFI.

### MDFI promoted CRC cell proliferation in vitro and CRC growth in vivo

**Figure 3.**
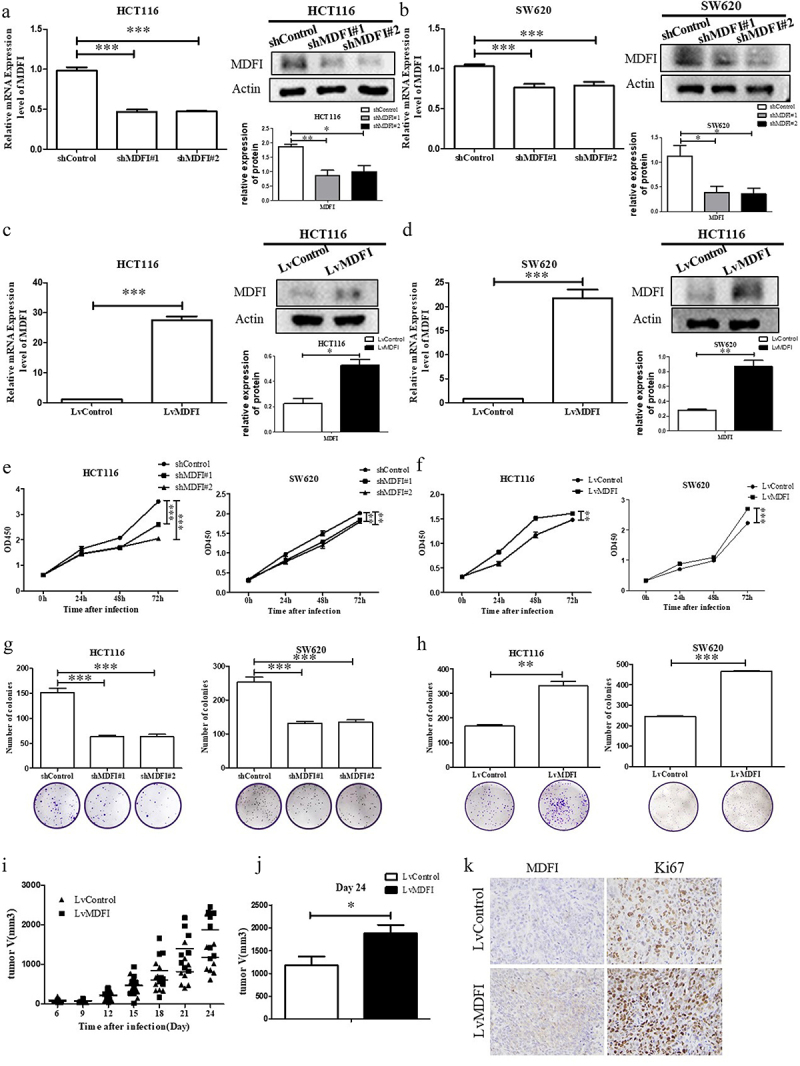
Effect of MDFI on CRC progression in vivo and in vitro. A. in HCT116 cells following shMDFI treatment, MDFI protein and mRNA expression were detected (mean with SEM, *n* = 3). B. in SW620 cells following shMDFI treatment, MDFI protein and mRNA expression were detected (mean with SEM, *n* = 3). C. in HCT116 cells following LvMDFI treatment, MDFI protein and mRNA expression were detected (mean with SEM, *n* = 3). D. in SW620 cells following LvMDFI treatment, MDFI protein and mRNA expression were detected (mean with SEM, *n* = 3). E. HCT116 and SW620 cells were treated with shMDFI, and cell viability was detected (mean with SEM, *n* = 3). F. HCT116 and SW620 cells were treated with LvMDFI, and cell viability was detected (mean with SEM, *n* = 3). G. HCT116 and SW620 cells were treated with shMDFI, and colony formation ability was detected (mean with SEM, *n* = 4). H. HCT116 and SW620 cells were treated with LvMDFI, and colony formation ability was detected (mean with SEM, *n* = 3). I. The volume of subcutaneous xenograft tumors from nude mice inoculated with HCT116 cells following LvMDFI treatment (mean with SEM, *n* = 8). J. at day 24, the volume of subcutaneous xenograft tumors isolated from nude mice inoculated with HCT116 cells following LvMDFI treatment (mean with SEM, *n* = 8). K. at day 24 subcutaneous xenograft tumors were isolated from nude mice inoculated with HCT116 cells following LvMDFI treatment, then MDFI and Ki67 protein were detected by immunohistochemistry.

To investigate the effect of MDFI on the progression of CRC in vitro and in vivo, we transfected MDFI-knockdown shRNA (shMDFI#1, shMDFI#2) and MDFI-overexpressing lentiviral vector plasmids (LvMDFI) into HCT116 and SW620 cells to construct MDFI-dysregulated CRC cells. The successful construction of CRC cell lines with MDFI knockdown or overexpression was confirmed at the mRNA and protein levels, respectively (Figure 3a–d). We subsequently explored the effect of the MDFI on the proliferation phenotype of CRC cells. The CCK8 assay showed that the survival rate of CRC cells significantly decreased after MDFI knockdown for 24–72 h, while the proliferation of CRC cells significantly increased after MDFI overexpression for 24–72 h (Figure 3e–f). Compared with that of control cells, the colony formation ability of CRC cells was significantly inhibited after MDFI knockdown for 48 h, while the result was the opposite after MDFI overexpression (Figure 3g–h), indicating the positive regulation of the MDFI gene on CRC progression in vitro. To verify the promoting effect of MDFI on the progression of CRC in vivo, we performed subcutaneous tumor loading on Balb/c nude mice. In nude mice transfected with lvMDFI CRC cells, tumor growth was faster than that in nude mice transfected with empty vectors (Figure 3i–j). Meanwhile, MDFI-overexpressing CRC tumors showed elevated Ki67 expression (Figure 3k). Based on these findings, MDFI may promote the proliferation of CRC cells both in vitro and in vivo.

### MDFI promotes cell cycle progression by upregulating CCND1 expression in CRC

**Figure 4.**
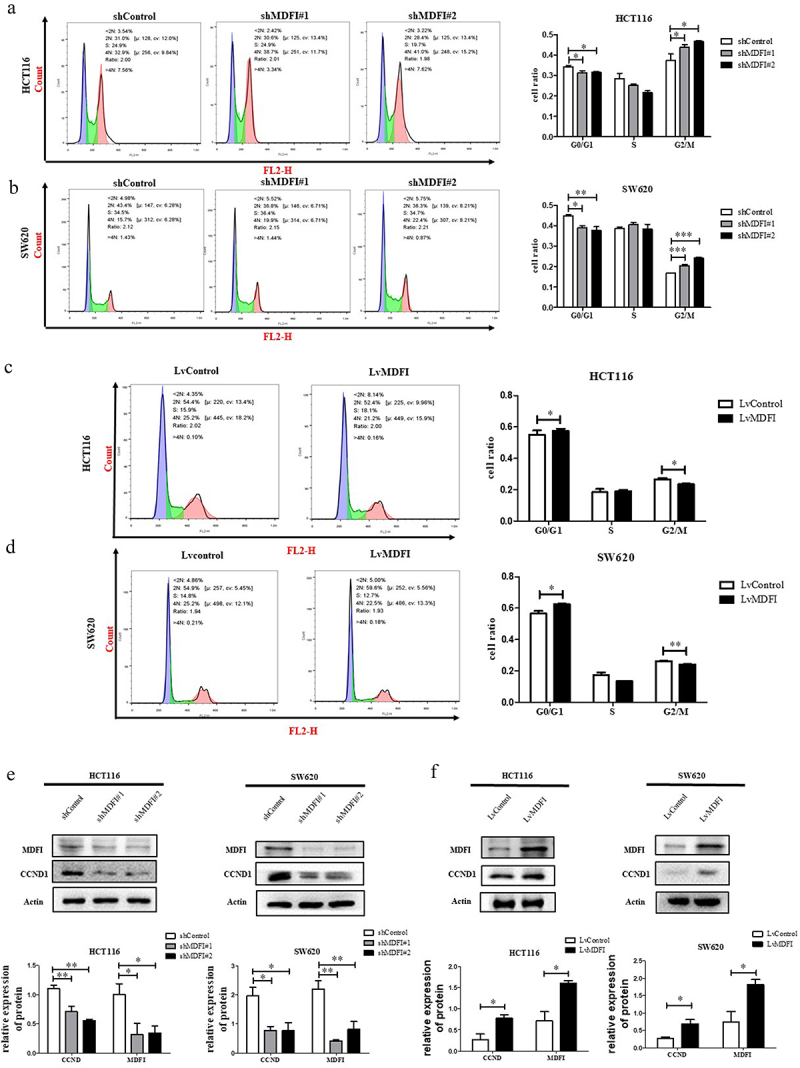
Effect of MDFI on the cell cycle in CRC cells A. HCT116 cells were subjected to shMDFI, after which the cell cycle distribution was detected via flow cytometry (mean with SEM, *n* = 3). B. SW620 cells were subjected to shMDFI, after which the cell cycle distribution was detected via flow cytometry (mean with SEM, *n* = 3). C. HCT116 cells were treated with LvMDFI, and the cell cycle distribution was detected by flow cytometry (mean with SEM, *n* = 3). D. SW620 cells were treated with LvMDFI, and the cell cycle distribution was detected by flow cytometry (mean with SEM, *n* = 3). E. CCND1 protein was detected in HCT116 and SW620 cells following shMDFI treatment (mean with SEM, *n* = 3). F. CCND1 protein was detected in HCT116 and SW620 cells following LvMDFI treatment (mean with SEM, *n* = 3).

To investigate the promoting effect of MDFI on CRC cell proliferation, we determined the cell cycle distribution of CRC cells with MDFI knockdown or overexpression. MDFI knockdown resulted in a significantly increased cell ratio of G2/M phase both in HCT116 (37.3 ± 3.2% in control vs 43.8 ± 1.4% and 46.7 ± 0.4%, respectively, in shMDFI#1 and shMDFI#2 group, p < .001) and SW620 cells (16.6 ± 0.2% in control vs. 20.5 ± 0.5% and 24.1 ± 0.4%, respectively, in shMDFI# 1 and shMDFI#2 group, p < .001) (Figure 4a–b). In contrast, the overexpression of MDFI caused a reduction in the proportion of HCT116 (26.7 ± 0.8% in the Lvcontrol group vs. 23.5 ± 0.4% in the LvMDFI group, p < .05) and SW620 (2.14 ± 0.26% in the Lvcontrol group vs. 0.64 ± 0.14% in the LvMDFI group, p < .001) cells in G2/M phase (Figure 4c–d). CCND1 is a cyclin protein that ensures the normal progression of mitosis.^23^ The expression levels of CCND1 were significantly decreased in CRC cells when MDFI was knocked down but increased after MDFI was overexpressed (Figure 4e–h), which confirmed the occurrence of cell cycle arrest induced by MDFI in CRC cells. Our study showed that MDFI accelerated the regular transition from the G2/M to G0/G1 phase to sustain CRC proliferation. Inhibition of MDFI expression induced G2/M phase arrest in CRC cells, thus blocking cell cycle progression and inhibiting CRC proliferation.

### MDFI was coexpressed with ITGB4 and LAMB3 and jointly promoted AKT pathway activation in CRC

**Figure 5.**
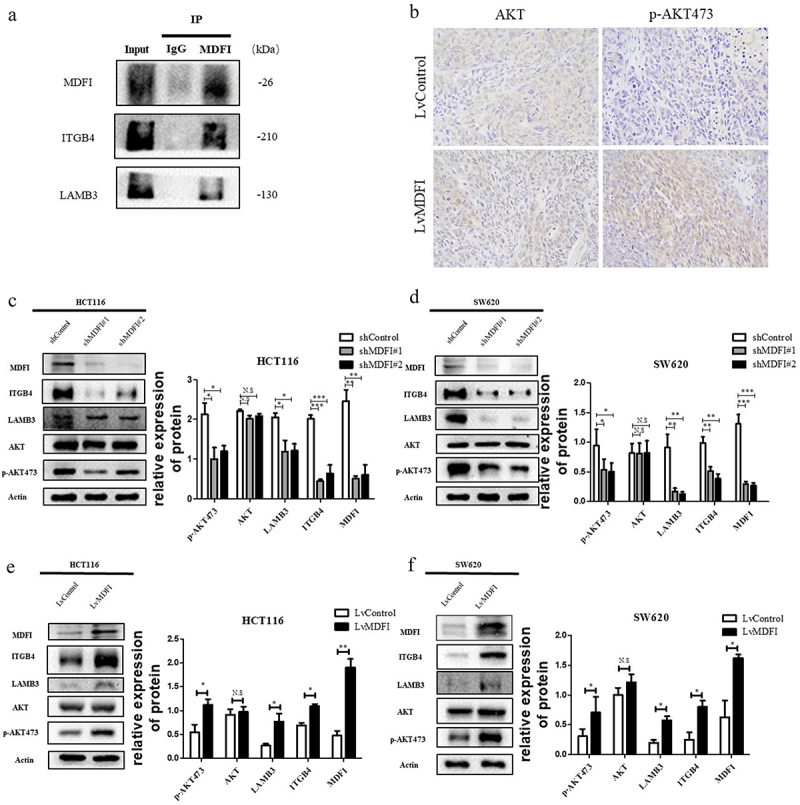
Effect of MDFI on the AKT pathway in CRC cells A. Co-immunoprecipitation was used to examine the direct interaction between MDFI and ITGB4/LAMB3. B. in HCT116 cells following LvMDFI treatment, AKT and p-AKT473 protein were detected by immunohistochemistry. C. HCT116 cells were subjected to shMDFI, and the levels of ITGB4, LAMB3, AKT and p-AKT473 proteins were detected by western blotting (mean with SEM, *n* = 3). D. in SW620 cells following shMDFI treatment, ITGB4, LAMB3, AKT and p-AKT473 proteins were detected by western blotting (mean with SEM, *n* = 3). E. in HCT116 cells following LvMDFI treatment, ITGB4, LAMB3, AKT and p-AKT473 proteins were detected by western blotting (mean with SEM, *n* = 3). F. in SW620 cells following LvMDFI treatment, ITGB4, LAMB3, AKT and p-AKT473 proteins were detected by western blotting (mean with SEM, *n* = 3).

Preliminary bioinformatic results indicate that MDFI plays a key role in regulating PI3K-AKT signaling in CRC cells. Additionally, MDFI gene expression was significantly correlated with ITGB4 and LAMB3, and MDFI had a direct interaction with ITGB4 and LAMB3. Immunoprecipitation indicated a physical link between MDFI and ITGB4 and LAMB3 in HCT116 cells (Figure 5a). In addition, MDFI-overexpressing HCT116 cells were used to construct a xenotransplantation model in nude mice, and compared with the control group, tumor tissues expressed significantly higher levels of p-AKT473, while the expression level of t-AKT (total AKT) was not significantly different. MDFI promoted AKT pathway activation (Figure 5b).

Several studies have demonstrated that AKT signaling accelerates the progression of CRC cells.^14^ In addition, ITGB4 and LAMB3 are important upstream activators of AKT, which is why we focused on the AKT signaling pathway. After MDFI knockdown, ITGB4, LAMB3 and p-AKT473 expression levels in HCT116 and SW620 cells were significantly decreased (Figure 5c–d). Compared with control cells, t-AKT expression was not significantly different (Figure 5c–d). The expression of ITGB4, LAMB3 and p-AKT473 was significantly upregulated after overexpression of MDFI (Figure 5e–f).

These results suggested that MDFI interacted directly with ITGB4 and LAMB3 to jointly induce AKT phosphorylation to promote the activation of the AKT signaling pathway and the progression of CRC.

### The AKT inhibitor MK2206 exerted an antitumor effect by inhibiting the activation of the AKT signaling pathway induced by MDFI in CRC

**Figure 6.**
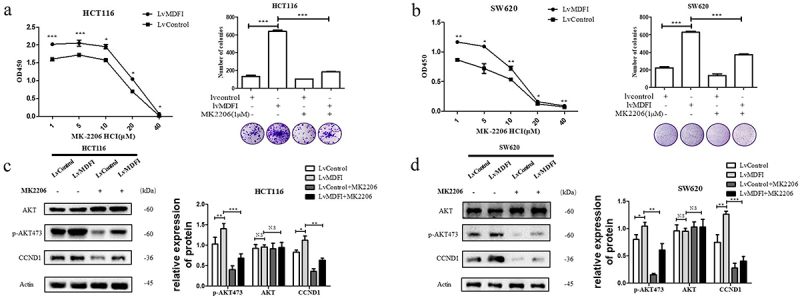
Effect of MK2206 on the activation of the AKT signaling pathway induced by MDFI in CRC cells A. Cell viability and colony formation ability were detected in control HCT116 cells and MDFI-overexpressing HCT116 cells following MK2206 treatment (mean with SEM, *n* = 3). B. Cell viability and colony formation ability were detected in control SW620 cells and MDFI-overexpressing SW620 cells following MK2206 treatment (mean with SEM, *n* = 3). C. AKT, p-AKT473 and CCND1 proteins were detected in control HCT116 cells and MDFI-overexpressing HCT116 cells following MK2206 treatment (mean with SEM, *n* = 3). D. AKT, p-AKT473 and CCND1 protein were detected in control SW620 cells and MDFI-overexpressing SW620 cells following MK2206 treatment (mean with SEM, *n* = 3).

Using MK2206, we confirmed the mechanistic effect of MDFI on CRC, and MK2206 is an AKT-specific inhibitor.^17^ Compared with that of HCT116 and SW620 cells without the treatment of MK2206, the survival rate of CRC cells decreased significantly after treatment with MK2206 for 24 h (Figure 6a–b). Subsequently, we examined why MK2206 inhibited MDFI-induced AKT activation. According to the western blotting results, the protein levels of p-AKT473 and CCND1 in the LvMDFI group were significantly decreased after treatment with 1 μM MK2206, while there was no significant difference in t-AKT (Figure 6c–d). These results indicated that MDFI induced the activation of the AKT pathway in HCT116 and SW620 cells, while MK2206 reversed the activation and inhibited CRC cell viability.

### The MDFI reduced chemotherapy sensitivity in CRC cells

Oxaliplatin and 5-fluorouracil (5-FU) are important components of systemic chemotherapy for CRC, and antitumor efficacy is mainly achieved by inhibiting thymidylate synthase to disrupt DNA replication and inhibit DNA damage repair.^24,25^ With the development of drug resistance in CRC, it is difficult to obtain continuous antitumor effects of chemotherapy drugs and overcome clinical drug resistance. The foregoing studies indicated that MDFI played an important role in regulating the malignant phenotype of CRC. To further investigate whether MDFI affects the chemotherapeutic sensitivity of CRC, we investigated the effect of MDFI on oxaliplatin and fluorouracil resistance by cytotoxicity and colony formation assays.

**Figure 7.**
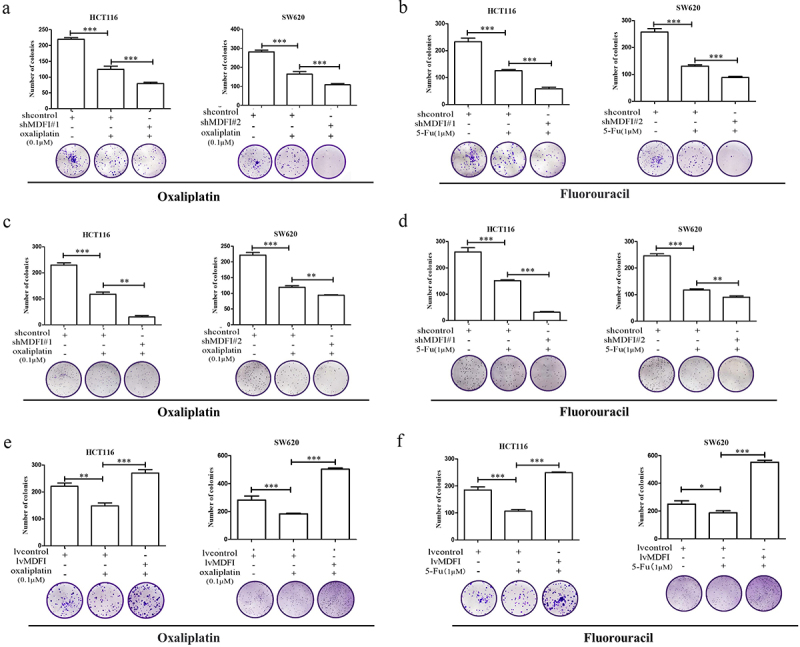
Effect of the MDFI on the chemotherapy sensitivity of CRC cells A. HCT116 and SW620 cells were treated with shMDFI#1 and oxaliplatin, and colony formation ability was detected (mean with SEM, *n* = 4). B. HCT116 and SW620 cells were treated with shMDFI#1 and 5-fu, and colony formation ability was detected (mean with SEM, *n* = 4). C. HCT116 and SW620 cells were treated with shMDFI#2 and oxaliplatin, and colony formation ability was detected (mean with SEM, *n* = 4). D. HCT116 and SW620 cells were treated with shMDFI#1 and 5-fu, and colony formation ability was detected (mean with SEM, *n* = 4). E. HCT116 and SW620 cells were treated with LvMDFI and oxaliplatin, and colony formation ability was detected (mean with SEM, *n* = 4). F. HCT116 and SW620 cells were treated with LvMDFI and 5-fu, and colony formation ability was detected (mean with SEM, *n* = 4).

Following treatment with oxaliplatin and 5-FU for 24 hours, the CRC cell survival rate decreased in a concentration-dependent manner. Compared to those in the control group, the proliferation levels in shMDFI#1 and shMDFI#2 decreased significantly, which was consistent with what was observed in SW620 cells. Compared with that of the control group, the colony formation ability of the shMDFI#1 and shMDFI#2 CRC cells was significantly decreased (Figure 7a–d). The proliferation level in the LvMDFI groups increased significantly compared with that in the control group, which was consistent with the SW620 results. Compared with that of the control group, the colony formation ability of MDFI-overexpressing CRC cells was significantly increased (Figure 7e–f).

These results suggested that MDFI could antagonize the inhibitory effects of oxaliplatin and 5-FU on CRC cell proliferation. Reducing the expression level of MDFI in CRC could effectively improve the chemotherapy effect of oxaliplatin and 5-FU in CRC.

## Discussion

Undoubtedly, CRC is one of the most aggressive and lethal forms of cancer in humans.^1^ The main task of CRC clinical research is to deeply analyze the potential mechanism of CRC occurrence and find more effective CRC molecular diagnostic and therapeutic targets. In recent years, some new technologies have been used to search for these targets, among which single-cell sequencing and PPIs have revolutionized cancer research.^26,27^ Using single-cell sequencing, Yuan Zhou et al. identified prevalent genomic alterations in the tumor stroma of CRC patients.^28^ Kennedy SA et al. discovered effective CRC molecular targets and their network in CRC cells by PPIs.^29^ In this article, we performed detailed analyses of single-cell sequencing data from the GSE144735 by PPIs and revealed that MDFI was a potential CRC therapeutic target. Single-cell sequencing and PPIs are increasingly being used in diagnostic and therapeutic target research, with a recent increase in the number of studies published. In studies by Tan YQ, Zhang Q and others, numerous genes were initially identified as tumor-associated genes.^30–32^ However, none of these studies investigated the underlying mechanism of these diagnostic and therapeutic targets. We took this issue into account; thus, our results could provide additional comprehensive information about diagnostic and therapeutic targets. To identify an effective diagnostic and therapeutic target, it has been of great interest to study the relationship between genes and tumor-specific characteristics. On the one hand, researchers can identify tumor-specific genetic alterations, gene effects on immune infiltration, gene effects on survival and gene effects on drug sensitivity by visualization and analysis of genes, samples, and clinical data^33–36^; on the other hand, researchers can identify gene effects on cell migration, invasion and proliferation by in vitro and in vivo experiments.^37,38^ In this study, TCGA expression and clinical database analysis and in vitro/vivo experiments were performed to further verify that MDFI was an effective CRC diagnostic and therapeutic target.

Available studies indicate that the MDFI is a novel biomarker for poor prognosis in tumors, including colorectal, brain, gastric lung and pancreatic tumors.^10,39–41^ Chen Mi and Sui Y et al. discovered that inhibition of MDFI attenuates proliferation in both studies.^10,42^ However, the direct regulatory mechanism of MDFI in tumors has not been elucidated. MDFI was originally cloned as a transcription factor in the MyoD family that interacts with MyoD by masking nuclear localization signals and preventing DNA binding, and it interferes with myogenic factor function.^9^ Chen CM and Kusano S et al. discovered that the primary localization of MDFI is in the cytoplasm, while the secondary localization seems to be in the nucleus; thus, MDFI seems to have effects other than interaction partners of transcription factors belonging to the MyoD family. In this article, we discovered that MDFI can directly interact with LAMB3 and ITGB4, and that MDFI can considerably increase the expression of ITGB4 and LAMB3. These findings indicate for the first time that MDFI plays a novel role in mediating tumor progression by directly affecting tumor-related mechanistic pathways.

Over the past few years, the role of ITGB4 and LAMB3 in tumor progression has begun to be uncovered.^16,18^ Chao Leng et al. discovered that anoikis susceptibility was significantly increased through inhibition of AKT signaling when ITGB4 was knocked down and that ITGB4-EGFR activated the focal adhesion kinase (FAK) and AKT signaling pathways in hepatocellular carcinoma.^18^ Zhehui Zhu et al. discovered that high LAMB3 protein expression was a marker for poor prognosis due to its role in advancing tumors via the AKT pathway.^16^ However, the regulators of the LAMB3 and ITGB4 proteins have still not been detected. Existing studies have shown that ITGB4 and LAMB3 expression is regulated by miRNAs and lncRNAs.^43–45^ In this study, we discovered that MDFI regulates the expression of LAMB3 and ITGB4 by binding directly to LAMB3 and ITGB4. Ching-Yi Chen et al. discovered that KCNF1 is a regulator of ECM-integrin interactions and positively regulates ITGB4 downstream signaling and that KCNF1 knockdown enhanced the polarized deposition of the basement membrane. They also found that the expression of ITGB4, LAMC1 and LAMC2 was reduced in cells with KCNF1 downregulation.^46^ We speculate that MDFI is also a regulator of ECM-integrin interactions. Furthermore, MDFI attenuates extracellular matrix deposition, thereby leading to an increase in the expression of LAMB3 and ITGB4 detected by Western blotting.

Chen Mi et al. discovered that the Wnt/β-catenin pathway is activated by MDFI in gastric cancer cells, which promotes their proliferation,^42^ and our results showed that MDFI mediates CRC malignancy by regulating the AKT pathway. In Chen Mi’s study, MDFI was shown to regulate glycolysis, which affects the proliferation of gastric cancer cells via the Wnt/β-catenin pathway. In this study, we discovered that MDFI promoted cell cycle progression and reduced chemotherapy sensitivity. Coant N et al. demonstrated that the AKT signaling pathway could accelerate cell cycle progression and cell proliferation in CRC in vitro and vivo.^14^ Hai Huang et al. discovered that cell cycle arrest at the G2/M phase can be regulated by the suppression of AKT phosphorylation in vitro.^47^ In our study, we observed that CCND1 played a key role in the promotion of cell cycle progression by MDFI. In addition, we found that CCND1 was the cell cycle protein most closely associated with MDFI through TCGA data analysis (Figure S3A). Shuohui Dong et al. discovered that CRC resistance to 5-FU is caused by AKT signaling, which is activated nonoxygen-dependently by reactive oxygen species.^48^ Ye Zhang et al. discovered that the resistance of CRC to oxaliplatin can be reversed through inhibition of the AKT pathway. Therefore, we confirmed that MDFI promotes the CRC cell cycle by activating the AKT pathway and that MDFI inhibits CRC chemotherapy sensitivity. Furthermore, analysis of human samples treated with fluorouracil or oxaliplatin from TCGA database revealed that high expression of MDFI was correlated with drug resistance (Figure S3B). ROC curve analysis revealed that the MDFI might be a biomarker for drug tolerance in patients with colorectal cancer (Figure S3C). The MDFI can be involved in CRC malignancy regulation by several pathways. The search for the direct targets that MDFI interacts with and the signaling pathways involved are the key strengths of the present study. This provides another direction for studying MDFI function.

Our study sheds light on the significance of the MDFI as a compelling therapeutic target in colorectal cancer (CRC). Through a comprehensive analysis of single-cell sequencing data and protein-protein interactions, we unveil that MDFI plays a pivotal role in CRC progression by orchestrating the AKT pathway. This study also unveils a novel function of MDFI by directly binding to LAMB3 and ITGB4, leading to the activation of the AKT pathway. Our findings expand our understanding of MDFI’s involvement in CRC and highlight its potential as a promising avenue for targeted CRC treatment. The identification of downstream pathways influenced by MDFI and a deeper exploration of its mechanisms of action represent promising directions for future research endeavors. By elucidating the complex role of MDFI in CRC, our study opens new doors toward more effective therapeutic strategies for this aggressive malignancy.

## Materials and methods

### Data processing

We downloaded GSE144735 scRNA-seq from the Gene Expression Omnibus (GEO) database. With marker- based annotations, we selected the epithelial cells by an annotation information table. Then, the ScaleDate function was used to transform the data linearly. Next, using the RunPCA function, we performed PCA on the scaled data. To cluster cells, we used the FindNeighbors and FindClusters functions.^49^ Finally, we performed dimensionality reduction with the UMAP algorithm to visualize and explore these datasets.

### Differentially expressed biomarker genes between normal and tumor tissues

We identified markers of a mild group compared to a severe group by the FindAllMarkers function (Seurat package). Differential analysis was carried out on genes with > 1 CPM in over 25% of cells.

### Gene enrichment analysis

With the help of the gseKEGG function (clusterProfiler package), we performed Kyoto Encyclopedia of Genes and Genomes (KEGG) pathway analysis of the differentially expressed biomarker genes for annotation.^50^

### Construction of the co-expression module

The 5000 genes were first evaluated for their usability. Here is the definition of adjacency matrix Amn:

Amn = |Smn|^β^

Amn = The contiguity of genes m and n, Smn = The Pearson correlation between gene m and gene n. β as the soft‐thresholding parameter (scale‐free R2 = 0.85). We then transformed the adjacency matrix into a topological overlap matrix (TOM). According to the TOM‐based dissimilarity measure, genes with high absolute correlation were divided into gene modules.

### Direct genetic association identification

We define the level of relationship. Level 1: gene A contacts directly with gene B, as confirmed by yeast two-hybrid identifying endogenous protein – protein interactions or published systematic screening efforts at the Center for Cancer Systems Biology. Level 2: gene A interacts indirectly with gene B by gene C, as confirmed by yeast two-hybrid assays identifying endogenous protein – protein interactions or published systematic screening efforts at the Center for Cancer Systems Biology. Level 3: gene A interacts indirectly with gene B by gene C and gene D, as confirmed by yeast two-hybrid assays identifying endogenous protein – protein interactions or published systematic screening efforts at the Center for Cancer Systems Biology.

### Validation of the genes that have the most direct connections

TCGA-COAD was used for further validation, Kaplan – Meier plotter was used for survival analysis, and the association between MDFI expression and tumor stage was evaluated by violin plot. We compared the expression of MDFI and LAMB3 and ITGB4 in the TCGA-COAD and CCLE datasets by using Pearson correlation.

### Cell culture

HCT116 and SW620 cells were obtained from Shanghai Cell Bank, Chinese Academy of Sciences (Shanghai, China). McCoy’s 5A medium was used to culture HCT116 cells (317–010, WISENT). RPMI 1640 medium was used to culture SW620 cells (350–007, WISENT).

### Lentivirus and shRNA

The MDFI-overexpressing lentivirus and shRNA-MDFI were obtained from Keygene Nanjing Kaiji Biotechnology Co., Ltd. (Nanjing, China). The sequences for target-specific shRNAs are as follows:

shMDFI#1: caCCGGAAGTTGCAGACGCAT;

shMDFI#2: ctGAACAGCATTGACCTCGAT;

The sequence of MDFI-overexpressing lentiviral particles of

p1: 5′-AGGTCGACTCTAGAGGATCCCGCCACCATGTACCAGGTGAGCGGCCAG-3′;

p2: 5′-TCCTTGTAGTCCATACCGGAGGAGAAGCAGAGCCCACAGCACTCCATG-3′.

### Western blot analysis

Cells were gathered and lysed in NP40 buffer (comprising 150 mM NaCl, 0.5% EDTA, 50 mM Tris, and 0.5% NP40) before being centrifuged at 12,000×g and 4°C for 15 minutes. Subsequently, either ten or twenty micrograms of total harvested protein were loaded and separated on an 8, 10, or 12% SDS-polyacrylamide gradient gel. The proteins were then transferred to polyvinylidene difluoride membranes and blocked with 5% nonfat milk at room temperature for 2 hours. Following this, the membranes were exposed to primary antibodies overnight at 4°C, succeeded by HRP-conjugated secondary antibodies at room temperature for 2 hours. After undergoing three washes in TBST, protein bands were visualized using an ECL chemiluminescence system from Bio-Rad, and the protein bands were quantified by using ImageJ. Each experiment was replicated three times.

### Colony formation assay

Cells were plated in cell culture dishes and incubated at 37°C in 5% CO2. The cells were treated, followed by incubation in medium alone for the next 6 days. After this 6-day period, colonies were fixed with 4% paraformaldehyde for 1 hour and consecutively stained with 0.5% crystal violet for 20 minutes. Subsequently, the number of colonies was enumerated, and photographs were captured. Each experiment was replicated three times.

### Cell cycle assay

The cell cycle distribution of diverse cell types was assessed using flow cytometry. Cells (approximately 1 × 106 cells per well) were collected after various treatments and fixed overnight in 70% ethanol at 4°C. Following fixation, the cells were centrifuged at 1,000 × g for 5 minutes to eliminate ethanol, washed, and stained with propidium iodide (PI) (10 μg/mL) and RNase A (100 μg/mL) at room temperature for 30 minutes. Propidium iodide signals were detected using a BD FACSCalibur flow cytometer (Becton – Dickinson, San Jose, CA, USA). The distribution of cells in different phases of the cell cycle was analyzed and quantified using FlowJo software (Tree star, San Carlos, CA, USA). In the cell cycle distribution figure, the blue, green, and red segments represent cells in G1 phase, S phase, and G2/M phase, respectively. Each experiment was replicated three times.

### Immunohistochemical

Samples were fixed using a 4% formaldehyde solution and subsequently embedded in paraffin. The paraffin-embedded system was then sectioned into 4-μm slices. For sample incubation at 4°C, MDFI (Santa Cruz), ki-67 (Abcam) AKT (CST) and p-AKT473 (CST) primary antibodies were applied, with an incubation time of 12 hours. Subsequently, the slices underwent incubation at room temperature using an HRP-conjugated secondary antibody for 1 hour. Detection of the samples was achieved through 3,3′-diaminobenzidine and hematoxylin staining methods. Each experiment was replicated three times.

### Coimmunoprecipitation (co-IP) assay

Cells were lysed with RIPA buffer (Beyotime, China). After preclearing with rabbit IgG for 2 h, lysates were immunoprecipitated at 4°C overnight with the indicated antibodies and Protein A/G PLUS-Agarose beads (Santa Cruz). Immunoprecipitated proteins were collected for Western blotting after three washes with lysis buffer.

### In vivo tumorigenesis assay

From Nanjing University’s Model Animal Research Center, BALB/c nude mice aged 4 weeks were obtained (Nanjing, China). To test tumorigenicity in vivo, mice were injected with one million HCT116 cells into the right infra-axillary dermis. The size of the tumors was measured every three days until the experiment ended. It was ensured that the mice were kept in a temperature-controlled and humidity-controlled environment free of specific pathogens. Each group consists of 8 mice.

### Statistical analysis

The statistical analysis was performed using GraphPad Prism 5.0 and SPSS Statistics 22. Differences between the two groups were compared by using an independent t-test, and a statistically significant difference was defined as *, *p* < .05; **, *p* < .01; ***, and *p* < .001.

## Supplementary Material

Supplemental Material

## Data Availability

The data underlying this article will be shared on reasonable request to the corresponding author, Junhua Wu (wujunhua@nju.edu.cn).
