# Supplementary material for: MDFI promotes the proliferation and tolerance to chemotherapy of colorectal cancer cells by binding ITGB4/LAMB3 to activate the AKT signaling pathway
Source: Cancer Biol Ther. 2024 Feb 20;25(1):2314324. doi: 10.1080/15384047.2024.2314324 (PMC10880501; doi:10.1080/15384047.2024.2314324)
Supplement: Supplemental Material [file KCBT_A_2314324_SM1758.zip › Figure S2.docx]

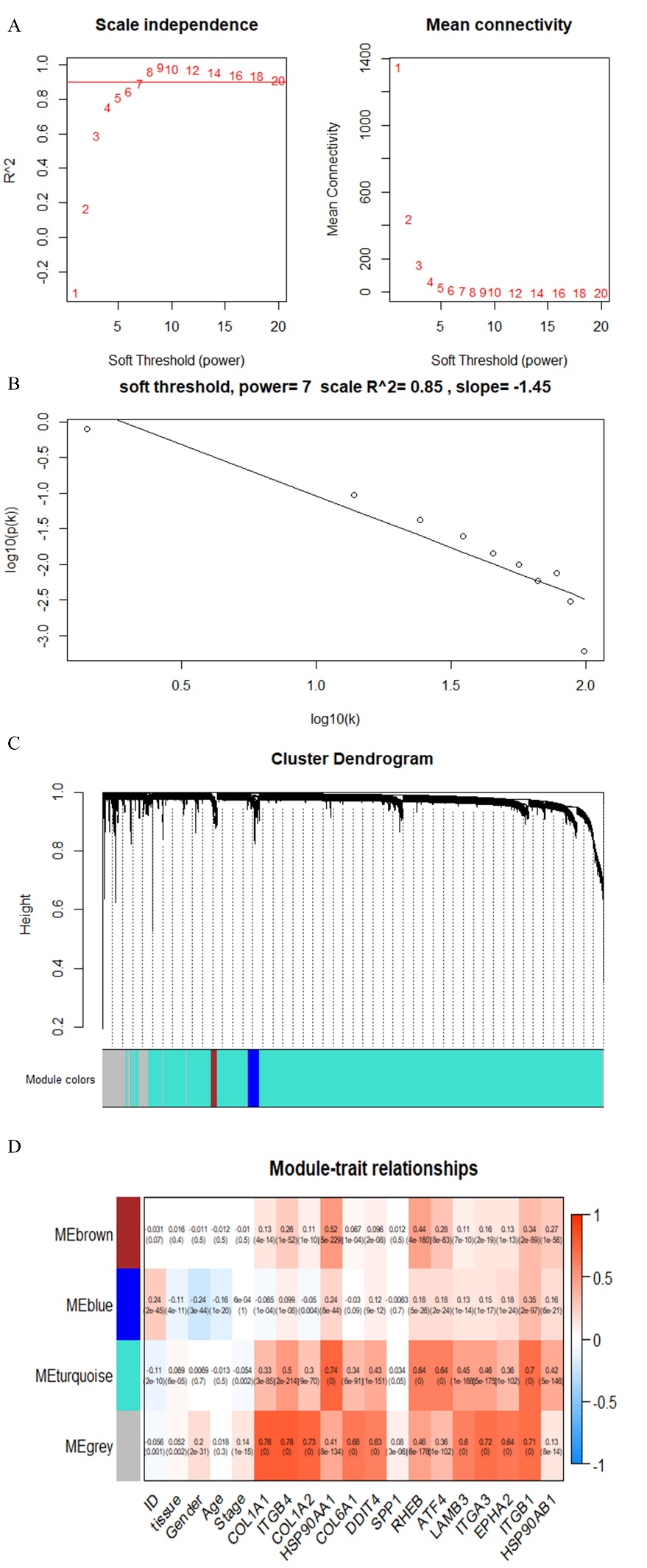


Figure S2 WGCNA identification of modules associated with the PI3K-AKT pathway genes and phenotype of colon cancer.

A. Analysis of the scale‐free fit index for various soft‐thresholding powers (β) and the mean connectivity for various soft‐thresholding powers B. Checking the scale‐free topology when β = 7. C. Dendrogram of all differentially expressed genes clustered based on a dissimilarity measure (1‐TOM). D. Heatmap of the correlation between module eigengenes and PI3K-AKT pathway genes of colon cells.
