# Supplementary material for: MDFI promotes the proliferation and tolerance to chemotherapy of colorectal cancer cells by binding ITGB4/LAMB3 to activate the AKT signaling pathway
Source: Cancer Biol Ther. 2024 Feb 20;25(1):2314324. doi: 10.1080/15384047.2024.2314324 (PMC10880501; doi:10.1080/15384047.2024.2314324)
Supplement: Supplemental Material [file KCBT_A_2314324_SM1758.zip › Figure S1.docx]

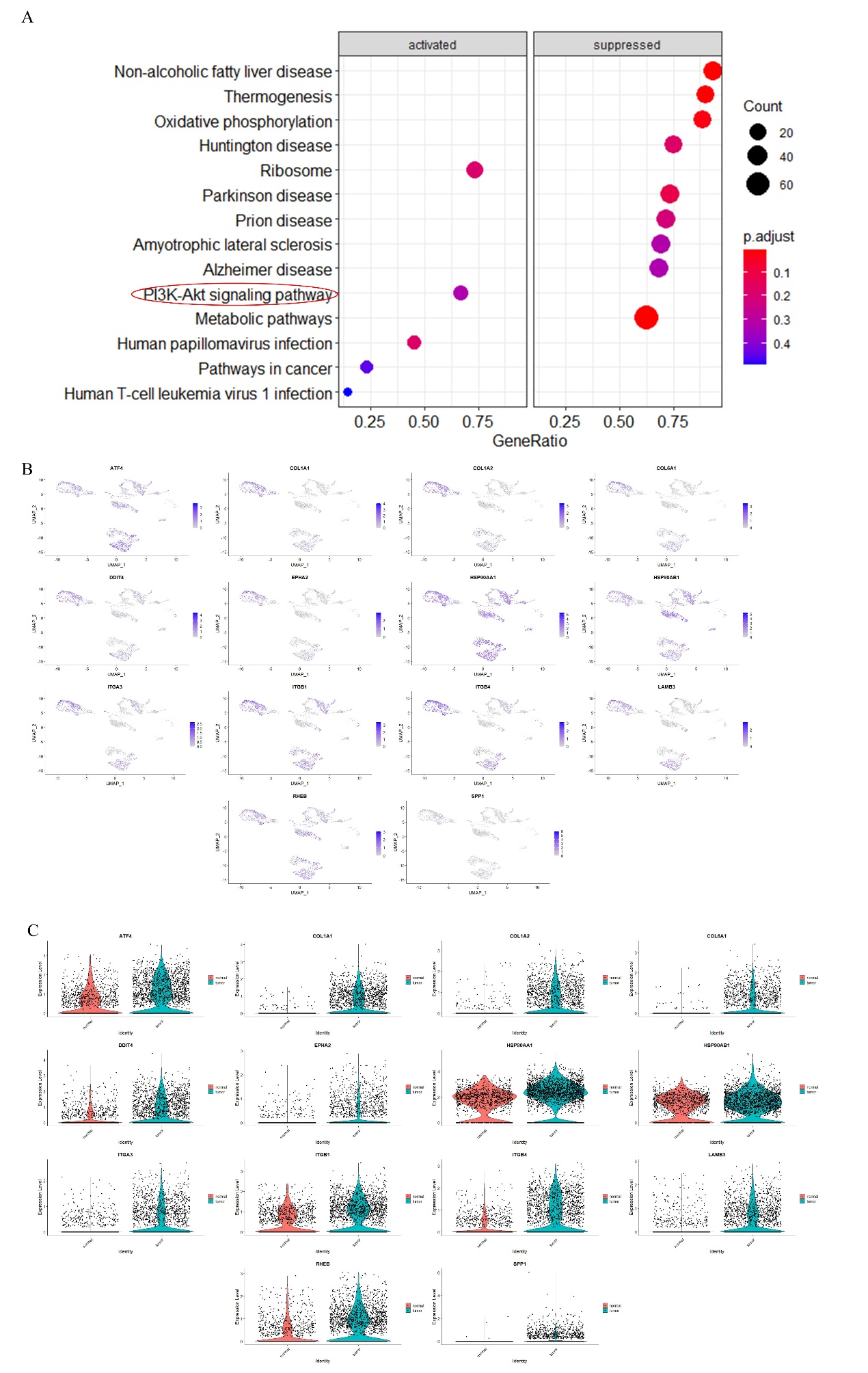


Figure S1 Comparison PI3K-AKT pathway of two epithelial cells, normal and tumor in GSE144735.

A. The KEGG analysis of Differential genes. B. UMAP plot shows the expression of PI3K-AKT pathway genes in tumor and normal colon cells. C. Violin plot shows the expression of PI3K-AKT pathway genes in tumor and normal colon cells.
