# Supplementary material for: MDFI promotes the proliferation and tolerance to chemotherapy of colorectal cancer cells by binding ITGB4/LAMB3 to activate the AKT signaling pathway
Source: Cancer Biol Ther. 2024 Feb 20;25(1):2314324. doi: 10.1080/15384047.2024.2314324 (PMC10880501; doi:10.1080/15384047.2024.2314324)
Supplement: Supplemental Material [file KCBT_A_2314324_SM1758.zip › Figure S3.docx]

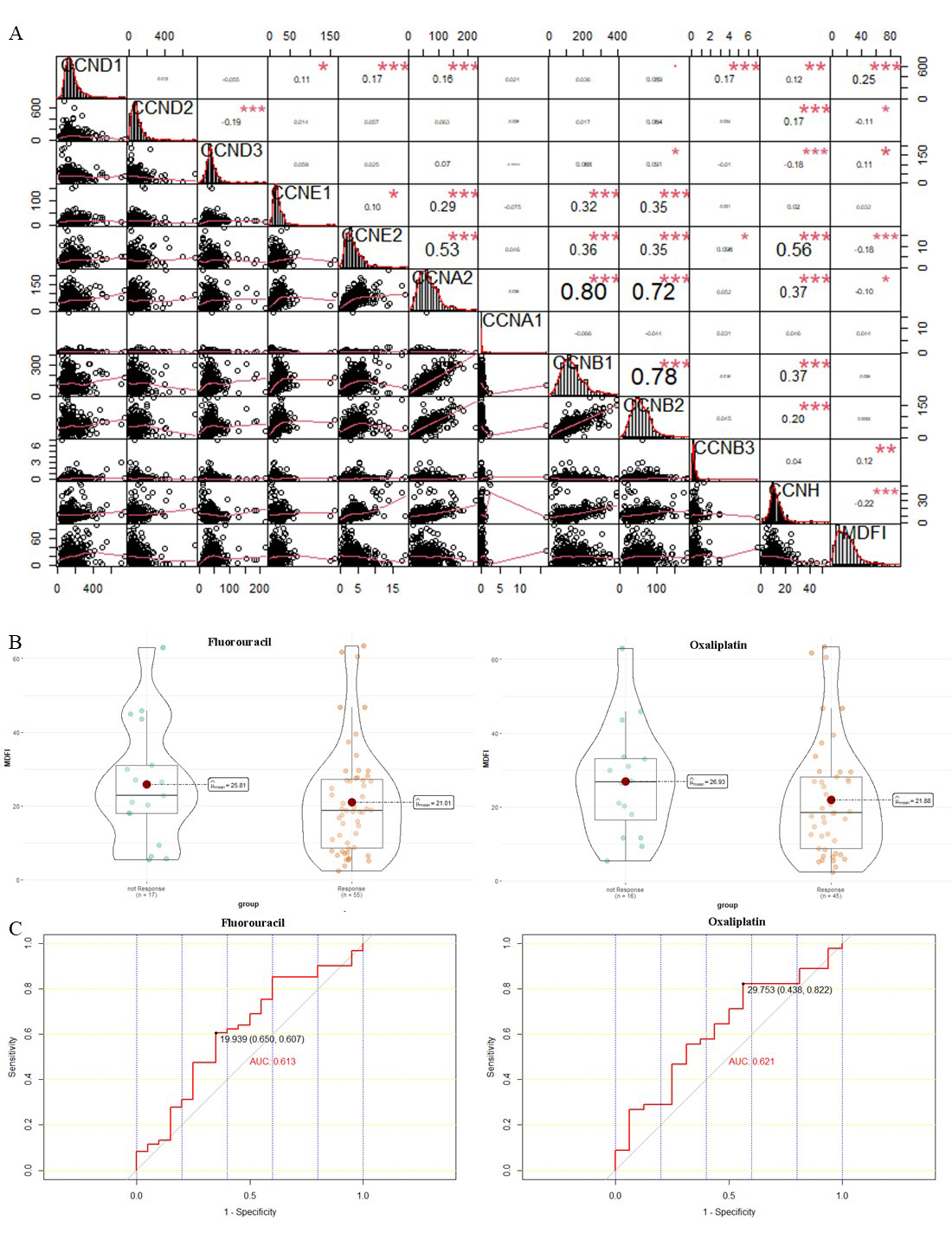


Figure S3 The Relationship between MDFI and Cell Cycle Proteins as well as Chemotherapy Resistance in Colorectal Cancer.

A. The correlation between tumor MDFI expression and the expression levels of cell cycle proteins such as CCND1, CCND2, CCND3, CCNE1, CCNE2, CCNA1, CCNA2, CCNB1, CCNB2, CCNB3, and CCNH in the TCGA-COAD database. B. In patients from the TCGA-COAD dataset treated with fluorouracil, the expression levels of MDFI in the response and non-response groups. C. ROC Curve of the Drug Response Prediction Model based on MDFI. The horizontal axis represents the False Positive Rate (FPR), and the vertical axis represents the True Positive Rate (TPR).
